# Rare Cases of Pediatric Vasoactive Intestinal Peptide Secreting Tumor With Literature Review: A Challenging Etiology of Chronic Diarrhea

**DOI:** 10.3389/fped.2020.00430

**Published:** 2020-08-05

**Authors:** Pai-Jui Yeh, Shih-Hsiang Chen, Jin-Yao Lai, Ming-Wei Lai, Cheng-Hsun Chiu, Hsun-Chin Chao, Shih-Hsin Chen, Ren-Chin Wu, Chao-Jan Wang, Chien-Chang Chen

**Affiliations:** ^1^Division of Pediatric Gastroenterology, Department of Pediatrics, Chang Gung Memorial Hospital, Linkou Branch and Chang Gung University College of Medicine, Taoyuan City, Taiwan; ^2^Division of Hematology and Oncology, Department of Pediatrics, Chang Gung Memorial Hospital, Linkou Branch and Chang Gung University College of Medicine, Taoyuan City, Taiwan; ^3^Department of Pediatric Surgery, Chang Gung Memorial Hospital, Linkou Branch and Chang Gung University College of Medicine, Taoyuan City, Taiwan; ^4^Division of Pediatric Infectious Diseases, Department of Pediatrics, Chang Gung Memorial Hospital, Linkou Branch and Chang Gung University College of Medicine, Taoyuan City, Taiwan; ^5^Department of Nuclear Medicine and Molecular Imaging Center, Chang Gung Memorial Hospital, Linkou Branch and Chang Gung University College of Medicine, Taoyuan City, Taiwan; ^6^Department of Pathology, Chang Gung Memorial Hospital, Linkou Branch and Chang Gung University College of Medicine, Taoyuan City, Taiwan; ^7^Department of Medical Imaging and Intervention, Chang Gung Memorial Hospital, Linkou Branch and Chang Gung University College of Medicine, Taoyuan City, Taiwan

**Keywords:** vasoactive intestinal peptide, diarrhea, children, tumor, WDHA syndrome, case report

## Abstract

Vasoactive intestinal peptide (VIP) secreting tumor (VIPoma) is a rare disease, presenting with profuse diarrhea, electrolyte imbalance, and possibly fatal outcome. The diagnosis and treatment are challenging, and no consensus guideline of management is available. The pediatric incidence remains unclear. This study comprises two pediatric case reports from a tertiary center and a literature-based case series investigating the characteristics among children. The two reported cases both presented with severe diarrhea and laboratory abnormalities, including electrolyte imbalance and elevated plasma VIP level. Case 1 received several imaging investigations, partial pancreatectomy, octreotide, and everolimus, reflecting her complicated and refractory course. Case 2 underwent total excision of suprarenal ganglioneuroblastoma, and the clinical response was significant. In both cases, varied degrees of symptomatic control, reduced plasma VIP level, and correction of electrolyte imbalance were achieved. A literature review-based case series analyzed 45 pediatric cases retrieved from the PubMed database until December 31, 2019. Demographics, clinical features, diagnostic modalities, treatments, and outcomes were presented.

## Introduction

Vasoactive intestinal peptide (VIP) secreting tumors (VIPoma) comprise several types of neuroendocrine tumor (NET), secreting VIP, which results in the “WDHA syndrome”: watery diarrhea, hypokalemia, and achlorhydria (1). Other presentations include hyperglycemia, metabolic acidosis, flushing, abdominal distention disproportionate to the nutritional state, and growth arrest (1–3). The estimated annual incidence is around one in 10 million people, yet the incidence of the pediatric population is unclear (3). Although pancreatic neoplasm represents the majority of adult VIPoma, the lesions among children are mostly extrapancreatic, including ganglioneuroma (GN), ganglioneuroblastoma (GNB), pheochromocytoma (PCC), and mastocytoma (2, 4). The reported age of onset of VIPoma ranges from 2 to 83 years (5). Plasma VIP level and imaging test are potential diagnostic tools, yet no consensus has been established. Tumor excision is the ideal treatment, but unresectable diseases rely on systemic therapy, including somatostatin analogs (SSAs), chemotherapy, molecular target therapy (MTT), and peptide receptor radionuclide therapy (PRRT) (3). Management remains challenging owing to the lack of optimal guideline and comprehensive survey of efficacy, especially for children (5).

## Case Reports

### Case 1

This 7-year-old girl presented with blood-tinged and mucoid diarrhea for around 2 months. The fecal consistency turned watery progressively, reaching over 10 times per day. Accompanied symptoms included intermittent periumbilical pain, nausea, episodes of non-bilious vomiting, poor appetite, and general weakness. Afebrile facial flushing was occasionally noted.

She was admitted to a tertiary hospital in Southern Taiwan initially. Fecal culture of virus, bacteria, and parasite yielded no growth. Levels of C-reactive protein (CRP), erythrocyte sedimentation rate (ESR), antinuclear antibody (ANA), and complement (C3 and C4) were normal. Abdominal computed tomography (CT) reported paralytic ileus without evidence of abscess. Broad-spectrum antimicrobial therapy received no expected response. Corticosteroids and mesalazine also failed as therapeutic trials regarding possible inflammatory bowel disease (IBD). Owing to complicated condition with unknown diagnosis, she was transferred to our hospital.

**Figure 1:**
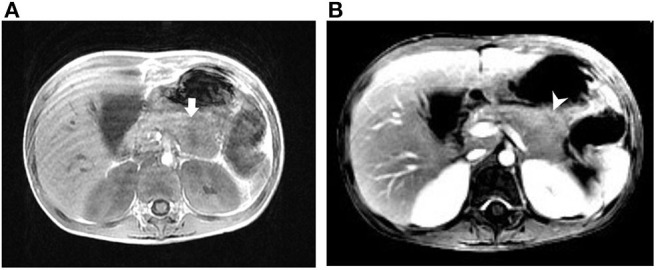
Magnetic resonance imaging of case 1. **(A)** T1-weighted image (T1WI) showing an ill-defined mass lesion at the pancreatic body. **(B)** T1WI with contrast showing the slight enhancement of the pancreatic lesion with central hyposignal. Arrow (white), pancreatic mass; arrowhead (white), central hyposignal of the lesion.

She appeared malnourished with distended abdomen. Her daily fecal output exceeded 5,000 ml/day (around 225 ml/kg/day, up to over 10,000 ml/day), despite of combined anti-diarrheal agents. Aggressive intravenous infusion and parenteral nutrition were administered. Laboratory examination showed elevated CRP (19.04 mg/L), hyponatremia (133 mEq/L), hypokalemia (2.6 mEq/L), and hypochloremia (96 mEq/L). Achlorhydria and metabolic acidosis were not evident. Stool osmotic gap was below 50 mOsm/kg, compatible with secretory diarrhea. Fecal calprotectin was 269 μg/g. Stool culture and toxin gene detection for *Clostridium difficile* were negative. Colonoscopy reported colitis with diffuse eruption of small, pinkish round nodules. Biopsies demonstrated mildly chronic inflammatory change without features of eosinophilic enterocolitis, cytomegalovirus colitis, or IBD. Her immune profile consisted of acceptable cellular proportion without deficiency of immunoglobulin. Levels of tumor markers, hormonal axis (thyroid and adrenal gland), tryptase, gastrin, calcitonin, chromogranin A, and catecholamine were all in normal ranges. Magnetic resonance cholangiopancreatography (MRCP) discovered a focally bulging, ill-defined mass lesion at the pancreatic body (Figure 1). In addition, plasma level of VIP was abnormally elevated (743.82 pg/ml), approximated to be 4-fold of the upper limit. Considering the presence of pancreatic VIPoma, intravenous octreotide was prescribed and tumor excision was arranged. Intraoperatively, a vaguely margined tumor of pancreatic body with encasement of splenic artery was found. Partial pancreatectomy (body) and splenectomy were performed. Microscopically, the mass comprised no architectural appearance of NET, yet some cells were positively stained by the VIP immunohistochemistry (IHC) agent.

Her fecal output reduced dramatically after the operation, as low as 400 ml/day. However, this headway merely lasted for a week, followed by relapse of diarrhea. The postoperative VIP level remained as high as 686.52 pg/ml. Repeated colonoscopy observed no specific evolution, except for mild progression of colonic nodules. Although the biopsies again failed to obtained clues of specific infection, neoplasm, or IBD, a few cells in the submucosa were positively stained in the VIP IHC stain. ^18^F-FDG PET scan revealed mild uptake at cervical lymph nodes, which also resided no tumor cells, confirmed by echo-guided biopsy. The ^68^Ga-DOTATOC PET/CT scan revealed physiologic uptake at pituitary gland, adrenal glands, and pancreatic uncinate process; unfortunately, no definite primary focus could be identified.

Because the operation and application of SSAs could not ameliorate her diarrheal predicament, everolimus was added. With the combination of everolimus and short-acting and long-acting octreotide, the fecal output reduced to around 1,000–3,000 ml/day. Plasma VIP level declined to 338.8 pg/ml after a total of 4 months of treatment. She resumed intake, and the daily required infusion of parenteral nutrition was minimized. Her fecal amount has been relatively controlled without occurrence of sepsis, shock, or acute abdominal complication.

### Case 2

This 11-month-old boy was admitted owing to intermittent afebrile watery diarrhea for 1 month, despite of formula adjustment and anti-diarrheal medications. His activity and urine output declined progressively. Laboratory examination revealed mild leukocytosis (11,200/μl), normal CRP level (1.99 mg/L), azotemia [blood urea nitrogen (BUN) 39.8 mg/dl, Cr 0.45 mg/dl], hyponatremia (118 mEq/L), hypokalemia (1.5 mEq/L), hypochloremia (93 mEq/L), hyperphosphatemia (1.5 mEq/L), and hypocalcemia (7.4 mEq/L).

**Figure 2:**
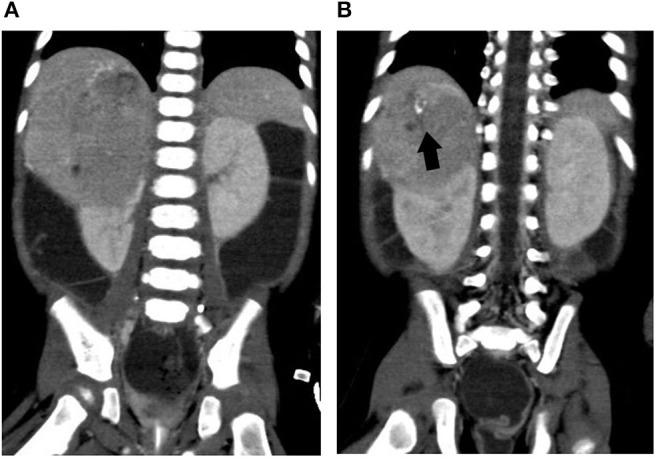
Abdominal computed tomography of case 2. **(A)** A huge right suprarenal mass lesion causing mass effect to adjacent liver and kidney. **(B)** A closer view of the part with calcification. Arrow (black), calcification in the tumor mass.

In the intensive care unit, he was rigorously resuscitated with intravenous fluid and electrolyte supplement. Abdominal sonogram reported a right suprarenal heterogeneous tumor with internal blood flow and calcification. Abdominal CT demonstrated a huge suprarenal lesion with necrotic component and mass effect to adjacent right kidney, possibly originating from suprarenal area or liver (Figure 2). Aldosterone (162 ng/dl), renin (20,984 ng/L), ACTH (98.9 pg/ml), cortisol (47.7 μg/dl), and urinary vanillylmandelic acid (VMA) (24 h, 12.9 mg/day) were all elevated, whereas the alpha-fetoprotein level was normal (4.6 ng/ml). Plasma VIP level was elevated (500.37 pg/ml). With the impression of VIPoma, he received operation on the fourth day of admission, with a large tumor sized 8.5 × 6.9 × 6.5 cm radically excised. The pathology report disclosed a GNB with weak positivity of VIP IHC stain in the GN component. Whole-body tumor scan discovered uptake over his right abdominal wall, neck lymph nodes, and spleen, suggestive of stage IV GNB.

Diarrhea subsided soon after the operation, facilitating a stabilization of fluid status and electrolyte level. He resumed intake 2 days after the surgery. The follow-up plasma VIP level was evidently reduced (156.94 pg/ml). With sustained resolution of symptoms, he was discharged on the 18th day of admission.

## Literature Review-Based Case Series

A literature review on PubMed database was performed using keywords “Vasoactive intestinal peptide secreting tumor” or “VIPoma” or “Verner-Morrison syndrome” or “VIP secreting tumor” and “children OR pediatric,” retrieving full-text case reports or case series published in English until December 31, 2019. The enrolled case qualified in the following requirements: (1) aged below 18 years; (2) relevant symptoms and laboratory results; (3) histopathologic or biochemistry (elevated VIP level > 190 pg/ml) evidence of VIPoma (4, 6); and (4) documentation of treatment and outcome. Case series with only statistical results and case reports without discrete depiction were excluded. Basic demographics, presentations, diagnostic processes, treatments, and outcomes were collected for analysis. The presence of VIP in tumor is defined as either positive extraction of VIP in lesion or positive result of VIP IHC stain.

Thirty-eight articles containing 61 cases were retrieved initially. Six patients with unclear duration of symptom and 10 patients with inadequately documented plasma VIP level (four cases with VIP level <190 pg/ml and six cases without VIP record) were excluded. In total, 45 cases were eligible for the final analysis (Table 1) (7–36).

**Table 1:** Demographics, presentations, tumor characteristics, intervention, and outcome.

**No**	**Year**	**Gender**	**AaD (Y)**	**OtD (M)**	**WD**	**H**	**A**	**Ac**	**Fl**	**VIP (pg/ml)**	**Site**	**Size (cm)**	**Pathology**	**Met**	**OP**	**Systemic treatment**	**Outcome**	**Ref**.
1	1975	F	5	30	Y	Y	–	–	–	292	M	–	GN	N	PE	N	CR	7
2	1976	M	1.4	5	Y	Y	–	–	–	450	Pv	–	GNB	N	TE	N	CR	8
3	1976	M	7.5	7	Y	Y	–	–	Y	3000	U	–	Unknown	N	Auto	N	Expired	9
4	1977	M	1.5	9	Y	Y	Y	–	–	645	Ne	4	GNB	N	TE	N	CR	10
5	1980	F	2.5	17	Y	Y	–	–	Y	287.3	Pv	8	GN	N	TE	N	CR	11
6	1980	M	0.7	4	Y	Y	–	–	Y	608	Sr	6.5	GNB	N	TE	N	CR	12
7	1980	F	3	4	Y	–	–	–	–	250	E	–	NF	N	Bx	N	Alive	13
8	1982	F	1.9	6	Y	Y	Y	–	–	840	Sr	7	GNB	N	TE	Y	CR	14
9	1982	F	3.9	26	Y	Y	Y	–	–	4900	Sr	6	GNB	Y	Auto	N	Expired	15
10	1982	F	1.5	2	Y	–	–	–	–	321.1	M	–	GNB	N	TE	Y	CR	16
11	1982	F	1.9	2.2	Y	Y	Y	–	–	520.5	Sr	5	GN	N	TE	N	CR	17
12	1983	M	7	12	Y	–	–	–	–	324.5	Sr	8	GN	N	TE	N	CR	18
13	1983	F	2	5	Y	Y	–	–	–	685	Sr	–	GNB	N	TE	N	CR	19
14	1983	M	1	0.5	Y	Y	–	–	–	1850	E	–	GN	Y	PE	Y	CR	19
15	1983	F	3	1.5	Y	Y	–	Y	–	554.3	Sr	4	GNB	N	TE	N	CR	20
16	1984	M	7	72	Y	Y	Y	–	–	682.8	Sr	4	GN	N	Auto	N	Expired	21
17	1984	M	3	20	Y	Y	–	–	–	317.7	Sr	4	GN	N	TE	N	CR	21
18	1984	F	1.8	3.6	Y	Y	Y	–	–	880	Sr	7	GNB	N	TE	N	CR	22
19	1984	F	1.9	6	Y	Y	Y	–	–	5200	Sr	7	GNB	N	TE	N	CR	22
20	1984	F	3.9	26.4	Y	Y	Y	–	–	4900	Sr	6	GNB	Y	PE	N	Expired	22
21	1984	F	1.6	14.4	Y	Y	Y	–	Y	940	Sr	5.3	GNB	N	TE	N	CR	22
22	1984	F	1.8	12	Y	Y	–	–	–	1000	M	–	NB	Y	PE	N	CR	22
23	1984	F	1.9	2.4	Y	Y	Y	–	Y	200	Sr	8.1	GN	N	TE	N	CR	22
24	1984	F	3.5	3.6	Y	Y	–	–	Y	590	M	10	GN	N	TE	N	CR	22
25	1984	F	1.5	4.8	Y	Y	Y	–	Y	900	Pv	3	GNB	N	TE	N	CR	22
26	1984	F	11	108	Y	Y	–	–	–	700	M	–	NB	Y	PE	N	CR	22
27	1984	F	2	4.8	Y	Y	–	–	–	2900	Sr	9.4	GNB	Y	PE	N	CR	22
28	1986	F	15	0.1	Y	Y	–	Y	–	2150	P	6	ICC	Y	TE	N	CR	23
29	1988	M	7	66	Y	Y	Y	–	–	1100	C	–	GNM	N	TE	N	CR	24
30	1988	F	3	3	Y	Y	–	–	–	1061.3	Pv	5	GN	N	TE	N	CR	25
31	1989	F	2.5	18	Y	–	–	–	–	1014	M	–	GNB	N	TE	Y	CR	26
32	1990	M	1.2	4	Y	Y	–	–	–	310.9	Sr	3	GNB	N	TE	N	CR	27
33	1993	F	5	4	Y	–	–	–	–	7000	L	–	MRT	Y	Bx	Y	Expired	28
34	2000	M	1.3	6	Y	Y	–	–	–	473.2	Ps	4	GN	N	TE	N	CR	29
35	2000	M	0.8	2.5	Y	Y	–	–	–	439.4	Sr	7	GNB	N	TE	N	CR	29
36	2000	M	2.2	16	Y	Y	–	Y	–	490.1	Sr	5	GN	N	TE	N	CR	29
37	2000	F	1.1	1.5	Y	Y	–	Y	–	517.1	Sr	5	GN	N	TE	N	CR	29
38	2003	F	1.6	4	Y	Y	–	–	–	3244.8	Ps	–	GNB	N	TE	Y	CR	30
39	2009	F	3	6	Y	Y	–	–	–	2805.4	Pv	5.8	GN	N	TE	N	CR	31
40	2013	F	1.2	6	Y	Y	–	–	–	679.5	Pv	–	GNB	N	TE	N	CR	32
41	2014	F	1.3	4	Y	Y	–	Y	–	247	Sr	4.5	GNB	N	TE	N	CR	33
42	2018	F	1.1	5	Y	Y	–	–	–	750.4	Ps	6.5	GNB	N	TE	Y	CR	34
43	2018	F	2	7	Y	–	–	–	–	246.7	Pv	10.5	GNB	Y	TE	Y	CR	34
44	2018	F	2	12	Y	Y	–	Y	–	6861.4	Sr	–	VIPoma	N	TE	Y	CR	35
45	2018	F	13	0.5	Y	Y	–	Y	Y	1105	P	4.5	VIPoma	N	TE	Y	CR	36

*A, achlorhydria; AaD (Y), age at diagnosis (year); Ac, acidosis; OtD (M), onset to diagnosis (month); Auto, autopsy; Bx, biopsy (any route); C, colon; CR, complete remission; E, extensive involvement; Fl, flushing; GN, ganglioneuroma; GNB, ganglioneuroblastoma; H, hypokalemia; ICC, islet cell carcinoma; L, liver; M, mediastinum; Met, metastases; MRT, malignant rhabdoid tumor; NB, neuroblastoma; Ne, neck; NF, neurofibroma; OP, operation; P, pancreas; PE, partial excision; Ps, pre-sacral; Pv, para/pre-vertebral; Sr, suprarenal; TE, total excision; U, unknown; VIPoma, vasoactive intestinal peptide secreting tumor; WD, watery diarrhea*.

### Characteristics, Presentations, and Diagnosis

Pediatric cases have been reported since the 1970s worldwide. Geographically, around 80% of our cases resided in the Eurasian area, whereas Japanese patients comprised the largest proportion in the Asia-Pacific group. The male-to-female ratio was 1:2.46. The age of diagnosis ranged from 0.7 to 15 years with an average of 3.3 years. The median interval of symptom onset to diagnosis was 5 months [interquartile range (IQR) 3.6–12 months), ranging from 0.1 to 108 months. All cases presented with diarrhea, whereas other presentations included facial flushing, fever, sweating, vomiting, abdominal pain, and hypertension. The leading laboratory abnormalities were hypokalemia (84.4%), achlorhydria (26.7%), and acidosis (15.6). The median plasma VIP level was 685 pg/ml (IQR 450–1,105 pg/ml), ranging from 200 to 7,000 pg/ml. The presence of VIP in tumor was documented in 20 cases (44%). Imaging modalities commonly applied included abdominal ultrasonography, CT, magnetic resonance imaging (MRI), intravenous pyelogram (IVP), and angiography. Nuclear medicine imaging [PET/CT scintigraphy, Octreoscan, and metaiodobenzylguanidine (MIBG) scintigraphy] was ever utilized on six patients. As for the location of lesion, the majority was extrapancreatic disease. Around half of the cases were found in the adrenal and suprarenal regions (46.7%), followed by paravertebral/prevertebral (15.6%) and mediastinum (13.3%). Only two cases (4.4%) developed pancreatic lesion. There were two cases manifested extensively with multiple organs involved: One developed prevertebral neoplasm extending from skull base to pelvis, and the other developed retroperitoneal lesion occupying left medial renal, para-aortic, and partial pancreas. Primary lesion of one case was unable to be determined even after autopsy, which revealed only pancreatic fibrosis. Another case presented with extensive colorectal ganglioneuromatosis, cured by total proctocolectomy 5 years after symptom onset. Except for cases without size record and immeasurable ones, the mean size of the primary tumor was 5.97 ± 1.96 cm. Nine (20%) cases had metastatic disease, with majorly invaded retroperitoneal and adjacent lymph nodes. As for histopathologic results, GN and GNB together accounted for 80% of the series, followed by neuroblastoma and neurofibroma. With the modification of guideline, some latest cases were termed as VIPoma with maturation grading.

### Treatment and Outcome

Thirty-four (75.6%) and six (13.3) patients underwent total and partial excision, whereas three patients received more than one operation. Autopsy was performed on two (4.4%) cases. Three (6.7%) patients underwent only exploratory operations for biopsy mostly owing to the unresectable size of huge tumor. SSA, chemotherapy, and radiotherapy were applied on two (4.4%), eight (17.8%), and five (11.1%) cases, respectively. An excluded case received sunitinib as adjuvant therapy, which was the only pediatric experience of molecular targeting therapy for VIPoma.

Most of the surgically treated patients gained adequate improvement, including cessation of diarrhea and substantial reduction of plasma VIP level. Forty (88.9%) cases remained alive, whereas 39 (86.7%) cases were documented with clinical improvement. Five (11.1%) mortality cases were reported, which resulted from sepsis, peritonitis, duodenal perforation, and profound diarrhea with severe hypokalemia. The average plasma VIP level for these five cases was 4,096.5 pg/ml, approximately 22-fold of the upper limit.

## Discussion

Since the first report of VIPoma in the 1970s, there have been approximately six review articles analyzing pediatric population (22, 25–27, 37, 38). In this series, 61 pediatric cases were retrieved before primary exclusion. Still, the incidence of children has not been clarified yet.

The mean age of diagnosis for VIPoma ranges from infancy to the eighties in different reports (5). In children, they are mostly diagnosed between 2 and 4 years, with the youngest case being 2 weeks old (25, 39). Reindl et al. (37) reported an age range of 2 months to 11 years (mean 2.6 years, median 1.9 years) in their series. Comparatively, we described a wider range, as 0.7–15 years (mean 3.3 years, median 2 years). Although some previous literatures disclosed no strong gender predominance or even male predominance, female preponderance was noted in this series (1:2.46), as reported in a previous pediatric series (1:1.7) (3, 37, 38, 40).

The interval of symptom onset to diagnosis varied, as the symptom often precedes diagnosis by 3–4 years (3). The range of interval approximated to a Japanese series (0.1–9 years), and the median interval also echoed to another series by Reindl et al. (22, 37). All patients presented with various severity and length of diarrhea. Achlorhydria developed in over 80% of patients in a Japanese series, but it seemed less described in this series (22). In addition to hypokalemia, several electrolytes could be concurrently affected. A case of hypocalcemic tetany was reported. Facial flushing appeared more prevalent in this series (17.8%) than in a previous all-aged report (8%) (4).

Consistently, this series demonstrated the predominance of extrapancreatic lesion in children, which is distinct to adult disease (2). Neoplasm originating from the retroperitoneal and mediastinal space comprised the majority, with a generally similar proportion of tumor location to previous reports (4). Only two pancreatic cases were identified, correlated to a recent review focusing on pancreatic NET (40). Among the measurable tumors, the mean size was 5.97 cm, ranging from 3 to 10.5 cm. A range of 1.2–5.9 cm was reported by Angelousi et al. in an all-aged series (3). Siddappa et al. (5) stated that the mean size of the primary lesion ranged from 4.4 to 5.4 cm. Overall, the lesion was likely to be at least 3 cm at diagnosis. Regarding metastasis, the pediatric rate was reported as 38% by Yamakuchi et al. among Japanese children in 1984 (22). Children encounter a lower metastatic rate owing to the minor proportion of primary pancreatic disease, supported by Siddappa et al. (5) (29% for all-aged neurogenic VIPoma).

Among asymptomatic children, plasma VIP level remains at a low and constant value of around 50 pg/ml (41). Several laboratory studies proposed a normal concentration of 0–190 pg/ml, adopted by many all-aged reviews (4, 6). VIP plays the crucial role in the pathophysiology and biochemical diagnosis of VIPoma (4). Judging from the significant response of VIP level, the utility of VIP in the confirmation and monitor of VIPoma is feasible for children. Even more, Reindl et al. (37) observed an association between VIP level and the severity of diarrhea.

In the microscopic aspect, a neuroblastic tumor with varied levels of differentiation constitutes the majority of pediatric disease. The International Neuroblastoma Staging System (INSS) and the Shimada system are applicable for prognostic perspectives. After the launch of WHO 2010 classification system for NETs and its update in 2017, the histological diagnosis followed this reporting format, which emphasizes a mitotic rate and a Ki-67 index (5). In addition to the neuroblastic tumor, some unique tumors were reported, including neurofibroma, intestinal ganglioneuromatosis, and rhabdoid tumor. Among the limited cases with IHC stain in the series, not all were positive for VIP. In a French series, only 23% of tumors (5/22) presented intra-tumor VIP IHC expression (38).

CT and MRI are fundamental tools for lesion localization and evaluation of disease extent. However, they sometimes yield inconclusive results, requiring second-line modalities (3). Endoscopic ultrasound provides excellent detection and guided sampling of lesion, but its application remains less prevalent among children (5). The performance of ^18^F-FDG PET scan and ^68^Ga-DOTATATE for NET are associated with the differentiation of tumor (42). Angelousi et al. (3) reported their sensitivity of ^18^F-FDG PET scan and ^68^Ga-DOTATATE as 30 and 79%. Similarly, Chen et al. (43) disclosed their sensitivity/specificity of ^18^F-FDG PET scan and ^68^Ga-DOTATOC as 41%/100% and 88%/100%, respectively. In this view, the diagnostic accuracy of ^68^Ga-DOTATOC for the detection of primary tumor sites was significantly higher than that of functional PET scan and Octreoscan. In this series, only six cases ever received functional imaging as evaluation, and only one case delineated adequate association of the uptake to the true lesion. In contrast, a case of ganglioneuromatosis of colon was reported to have positive uptake at the adrenal area; another case underwent pancreatoduodenectomy, because the initial scintigraphy indicated abnormalities of the pancreatic head, yet the final lesion was found to reside in the adrenal gland (24, 44). Although recent researches recommended ^68^Ga-labeled somatostatin analogs with PET/CT as an initial diagnostic tool, the experience for children remains scanty (5, 39).

Patients may require multimodality treatment to achieve disease control, including three levels: symptomatic, curative, and palliative (5). Generally, surgery is regarded as a standard treatment with potential of cure. However, Angelousi et al. (3) challenged this with a low (18%) rate of sustained disease-free status after the primary surgery in their series. In comparison, efficacy of surgery for children seems better. Almost 90% of cases underwent at least one excisional surgery, whereas 60% achieved complete resolution of symptom by surgery alone. In a previous French pediatric study, the surgical rate and diarrhea cessation rate were 62.5 and 100%, respectively (38). In another pediatric review, the surgical cure rate approached 84.2% (48/57) (37). Therefore, curative surgery for resectable disease seems to deserve consideration if feasible.

Prior literatures have underscored the role of non-surgical treatment. SSA is the mainstay of medical treatment, which could attain up to 83% of sustained response in all-aged study (5). SSA binds to the somatostatin receptors, facilitating the regulation of hormone secretion and the inhibition of tumor growth (1). Yet the application was less described in children. A possible explanation could be the preponderance of neuroblastic tumor, leading to a differed strategy to pancreatic ones in adults (39). All these cases with combination therapy have received preceding or concurrent surgeries. An excluded case with pancreatic lesion was treated with distal pancreatectomy, SSA, chemotherapy, and eventually sunitinib as MTT. Although this patient did not achieve complete remission, this was the only pediatric case using MTT (45). Everolimus and sunitinib are two promising MTTs as advanced therapy, participating as mammalian target of rapamycin (mTOR) inhibitor and vascular endothelial growth factor (VEGF) pathway inhibitor, respectively. These MTT demonstrate certain potential on metastatic diseases while sustaining a better progression-free survival (1). Successful treatment with sunitinib for adult has been reported, and the ENETS guideline has approved everolimus as a therapeutic option (46, 47). Still, pediatric experience of these novel agents is still scarce.

Among all-aged surveys, the median survival ranged from 70 to 96 months, whereas the mortality rate was 10 and 20% in two reviews (3, 5, 39, 40). Including this study, three larger pediatric series disclosed an approximate mortality rate of 11–13% (1989, 11.9%; 2004, 12.3%; and 2020, 11.1%) (26, 37). The survival rate up to 97% was found among children with neuroblastic VIPoma who underwent surgical treatment (26).

Owing to the retrospective design of the study, the analysis faced limitations including the presence of missing data, heterogeneity of reporting, varied diagnostic approaches, and the influence of evolution regarding novel imaging technique and medication.

## Conclusion

VIPoma is a rare disease resulting in profuse diarrhea and possibly lethal complications. Neuroblastic neoplasm with VIP secreting behavior comprises the majority of pediatric disease, leading to substantial differences to adults. Curative surgery is the ideal option for resectable tumor, although the efficacy of systemic treatment and novel agent for children requires more experience and evidence. Early recognition of this rare differential diagnosis may lead to better outcomes.

## Data Availability Statement

The raw data supporting the conclusions of this article will be made available by the authors, without undue reservation.

## Ethics Statement

Written informed consent was obtained from the individual(s), and minor(s)' legal guardian/next of kin, for the publication of any potentially identifiable images or data included in this article.

## Author Contributions

Diagnosis and management of the patients were carried out by C-CC, S-HsiaC, M-WL, H-CC, C-HC, S-HsinC, C-JW, J-YL, and R-CW. Writing of the manuscript was performed by P-JY and C-CC. Data analysis and interpretation were carried out by P-JY and C-CC. Critical evaluation and revision of the manuscript were performed by C-CC. All authors contributed to the article and approved the submitted version.

## Conflict of Interest

The authors declare that the research was conducted in the absence of any commercial or financial relationships that could be construed as a potential conflict of interest.
